# Preclinical Evaluation of ^99m^Tc-Labeled GRPR Antagonists maSSS/SES-PEG_2_-RM26 for Imaging of Prostate Cancer

**DOI:** 10.3390/pharmaceutics13020182

**Published:** 2021-01-30

**Authors:** Ayman Abouzayed, Sara S. Rinne, Hamideh Sabahnoo, Jens Sörensen, Vladimir Chernov, Vladimir Tolmachev, Anna Orlova

**Affiliations:** 1 Department of Medicinal Chemistry, Uppsala University, 751 23 Uppsala, Sweden; ayman.abouzayed@ilk.uu.se (A.A.); sara.rinne@ilk.uu.se (S.S.R.); hamideh.sabahnoo@ilk.uu.se (H.S.); 2 Division of Radiology and Nuclear Medicine, Department of Surgical Sciences, Uppsala University, 751 85 Uppsala, Sweden; jens.sorensen@pet.uu.se; 3 Department of Nuclear Medicine, Cancer Research Institute, Tomsk National Research Medical Center Russian Academy of Sciences, Tomsk 634009, Russia; chernov@tminc.ru; 4 Research Centrum for Oncotheranostics, Research School of Chemistry and Applied Biomedical Sciences, Tomsk Polytechnic University, Tomsk 634050, Russia; vladimir.tolmachev@igp.uu.se; 5 Department of Immunology, Genetics and Pathology, Uppsala University, 752 37 Uppsala, Sweden; 6 Science for Life Laboratory, Uppsala University, 752 37 Uppsala, Sweden

**Keywords:** prostate cancer, gastrin-releasing peptide receptor antagonist, technetium-99m, single-photon emission computed tomography, RM26

## Abstract

Background: Gastrin-releasing peptide receptor (GRPR) is an important target for imaging of prostate cancer. The wide availability of single-photon emission computed tomography/computed tomography (SPECT/CT) and the generator-produced ^99m^Tc can be utilized to facilitate the use of GRPR-targeting radiotracers for diagnostics of prostate cancers. Methods: Synthetically produced mercaptoacetyl-Ser-Ser-Ser (maSSS)-PEG_2_-RM26 and mercaptoacetyl-Ser-Glu-Ser (maSES)-PEG_2_-RM26 (RM26 = d-Phe-Gln-Trp-Ala-Val-Gly-His-Sta-Leu-NH_2_) were radiolabeled with ^99m^Tc and characterized in vitro using PC-3 cells and in vivo, using NMRI or PC-3 tumor bearing mice. SPECT/CT imaging and dosimetry calculations were performed for [^99m^Tc]Tc-maSSS-PEG_2_-RM26. Results: Peptides were radiolabeled with high yields (>98%), demonstrating GRPR specific binding and slow internalization in PC-3 cells. [^99m^Tc]Tc-maSSS-PEG_2_-RM26 outperformed [^99m^Tc]Tc-maSES-PEG_2-_RM26 in terms of GRPR affinity, with a lower dissociation constant (61 pM vs 849 pM) and demonstrating higher tumor uptake. [^99m^Tc]Tc-maSSS-PEG_2_-RM26 had tumor-to-blood, tumor-to-muscle, and tumor-to-bone ratios of 97 ± 56, 188 ± 32, and 177 ± 79, respectively. SPECT/CT images of [^99m^Tc]Tc-maSSS-PEG_2_-RM26 clearly visualized the GRPR-overexpressing tumors. The dosimetry estimated for [^99m^Tc]Tc-maSSS-PEG_2_-RM26 showed the highest absorbed dose in the small intestine (1.65 × 10^−3^ mGy/MBq), and the effective dose is 3.49 × 10^−3^ mSv/MBq. Conclusion: The GRPR antagonist maSSS-PEG_2_-RM26 is a promising GRPR-targeting agent that can be radiolabeled through a single-step with the generator-produced ^99m^Tc and used for imaging of GRPR-expressing prostate cancer.

## 1. Introduction

Gastrin-releasing peptide receptors (GRPR) are 7-transmembrane domains containing G-protein coupled receptors that are endogenously expressed in the pancreas and to a lower extent in other organs such as the stomach and the brain [1]. GRPR activated with an endogenous ligand, the gastrin-releasing peptide, regulates numerous functions of the gastrointestinal and central nervous systems, including the release of gastrointestinal hormones, smooth muscle cell contraction, and epithelial cell proliferation. GRPR overexpression is found in many malignancies, including prostate, breast, and colon cancers [2]. GRPR signaling is important for the growth, invasion, migration, and progression of prostate cancer [3], which is the second most commonly diagnosed cancer in men and is the cause of many cancer-related deaths [4].

Small prostate-specific membrane antigen (PSMA) targeting agents demonstrated the power of radionuclide-based theranostic approaches for prostate cancer [5]. However, low Gleason grade tumors have low PSMA expression, often on the luminal side of the glandular structures, and PSMA expression is lost in very advanced, de-differentiated prostate cancer, such as neuroendocrine prostate cancer. Therefore, there is a niche for other targeting theranostic agents in the clinic. GRPR overexpression is found in 63–100% of primary prostate tumors and in more than 50% of lymph node and bone metastases [6,7,8]. The expression of GRPR was found to be higher in the earlier stages of prostate cancer, and this overexpression is androgen-dependent [9,10,11]. GRPR expression in the prostate is largely limited to the malignant prostate cells, with the majority of benign and hyperplastic prostate tissue being GRPR negative [12].

The aforementioned information establishes GRPR as an important target for prostate cancer imaging and therapy. Several GRPR-targeting ligands have been developed and evaluated over the years [13]. The activation of GRPR triggers physiological responses [14], stimulates cell proliferation and growth [15], and downregulates the receptors’ expression [16]. Therefore, recent research and development of GRPR-targeting probes is more focused on GRPR antagonists than on agonists [17]. Radiolabeled GRPR antagonists performed equal to or even better than agonists in vivo [18,19].

GRPR antagonists that are based on d-Phe-Gln-Trp-Ala-Val-Gly-His-Sta-Leu-NH_2_ (RM26 [13]) have been developed and evaluated over the years showing high affinity towards GRPR and favorable pharmacokinetics [20]. In several studies, RM26 was conjugated to different chelators using a polyethylene glycol linker (PEG), and radiolabeled with various radionuclides for imaging and therapeutic purposes [20]. More recently, [^68^Ga]Ga-NOTA-PEG_3_-RM26 has been evaluated in early-phase clinical studies [21,22], demonstrating high safety and efficiency in the detection of primary prostate cancer and its metastases [19]. The radiolabeled GRPR antagonist [^68^Ga]Ga-NOTA-PEG_3_-RM26 also performed better than an agonist [^68^Ga]Ga-BBN when compared in a clinical study [19].

Positron emission tomography (PET) and single-photon emission computed tomography (SPECT) have been in use for molecular imaging for several decades. While PET scans can be preferred to SPECT scans because of better resolution, SPECT imaging is still used more than PET imaging worldwide, owing to its lower cost and the wider availability of SPECT-suitable radionuclides. In Europe hospitals operate 3.5-fold more SPECT cameras than PET ones [23]. Also, the advances made in SPECT technology, such as the cadmium-zinc-telluride (CZT) SPECT cameras, have significantly improved SPECT spatial resolution and sensitivity and offered accurate absolute tracer uptake quantification comparable to PET/CT [24]. The most commonly used SPECT radionuclide is ^99m^Tc (half-life of 6 h). The optimal energy of emitted photons for imaging, its wide availability, and the low costs of ^99^Mo/^99m^Tc generators facilitated its frequent use in diagnostic imaging. Several GRPR antagonists were therefore developed to be radiolabeled with ^99m^Tc to provide radiotracers simple in preparation for clinical use [25,26].

It has been observed that biodistribution and targeting properties of bombesin analogs are strongly dependent on the chelator used for labeling [20]. Modification of the chelator causes a re-distribution in charge and lipophilicity of the labeled peptide affecting its affinity and off-target interactions in vivo. Unfortunately, the effect of such modifications on biodistribution is difficult to predict a priori. This necessitates in vivo evaluation of a variety of different conjugates to find the best one.

Surprisingly, in vivo evaluation of mercaptoacetyl-containing peptide-based chelators for ^99m^Tc-labeling of GRPR antagonists has not been published so far. It has been demonstrated that the use of mercaptoacetyl-tri-glycine (MAG3, maGGG) enables stable labeling of an agonistic analog with preserved affinity [27]. However, follow-up in vivo evaluation has not been reported. Meanwhile, this type of chelator is attractive as it permits the production of whole conjugates by peptide synthesis. The use of mercaptoacetyl-containing peptide based chelators enables the formulation of single-vial kits for labeling [28], which simplifies clinical translation. Furthermore, the use of amino acids with different side-chains makes it possible to fine-tune the biodistribution properties of labeled proteins [29,30,31]. In our studies on labeling of HER2-targeting affibody, we have found that the use of serine and glutamate-containing variants enable more stable complexes of ^99m^Tc than the use of maGGG. For example, the use of serine-containing chelators instead of glycine-containing resulted in an appreciable decrease of activity uptake in the salivary gland and stomach, which indicated a reduced release of free technetium in vivo [29,30]. The reduction of the uptakes was proportional to the degree of substitution and was the most pronounced in the case of mercaptoacetyl-Ser-Ser-Ser (maSSS) chelator. The use of glutamate-based chelators had an even stronger effect [29,31]. However, a review of the literature [20] and our previous data [32,33] show that an increase of a negative charge of the chelator results in the decrease of the affinity of bombesin analogs to GRPR. Therefore, we considered that multiple incorporations of glutamates into the chelator would be undesirable, but the incorporation of a single one might increase stability without a dramatic decrease in affinity. Following this logic, we designed two GRPR antagonists that were based on RM26, linked via a PEG_2_ to a peptide-based chelator that is either formed by mercaptoacetyl-Ser-Ser-Ser (maSSS-PEG_2_-RM26) or by mercaptoacetyl-Ser-Glu-Ser (maSES-PEG_2_-RM26), to be suitable for direct labeling with ^99m^Tc. The main aim of this study was to provide a radiotracer that will be simple to radiolabel with ^99m^Tc and to use in clinics for imaging GRPR-overexpressing prostate cancers.

## 2. Materials and Methods

**Figure 1:**
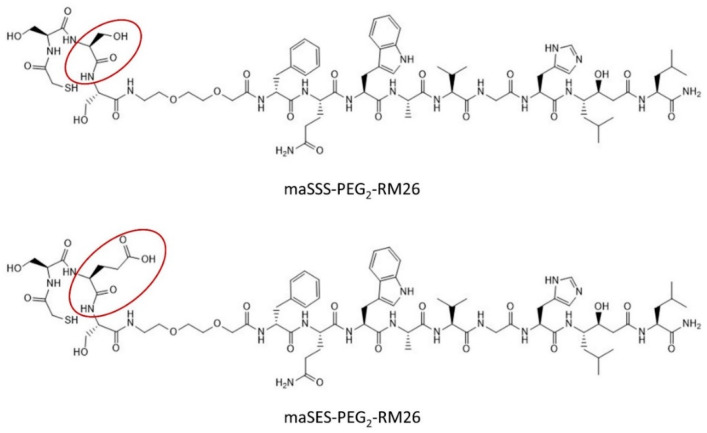
Structures of maSSS-PEG_2_-RM26 and maSES-PEG_2_-RM26.

Peptides maSSS-PEG_2_-RM26 and maSES-PEG_2_-RM26 (shown in Figure 1) were synthesized by Pepmic Co., Ltd. (Suzhou, China) according to our design. Prostate carcinoma cell line PC-3 (GRPR positive) was purchased from ATCC (Manassas, VA, USA) and maintained according to ATCC recommendations in RPMI-1640 media. Media supplements (fetal bovine serum, penicillin-streptomycin (100 IU/mL penicillin, 100 µg/mL streptomycin), 2 mM L-glutamine and trypsin-EDTA solution for cell detachment were purchased from Biochrom AG (Berlin, Germany). Technetium-99m was obtained as [^99m^Tc]NaTcO_4_ by elution from a ^99^Mo/^99m^Tc generator (Mallinckrodt Inc., St. Louis, MO, USA). The radioactivity content in cells and organs were measured using the 2480 Wizard^2TM^ gamma counter (PerkinElmer, Waltham, MA, USA).

### 2.1. Radiolabeling, Stability, and Octanol-Water Distribution Coefficient

Labeling of maSSS-PEG_2_-RM26 and maSES-PEG_2_-RM26 (two-vial protocol) was performed using freeze-dried labeling kits according to the previously described method [34]. Briefly, 10 µg of either peptide dissolved in phosphate-buffered saline (PBS) (5 mg/mL) was added to a freeze-dried kit (5 mg of gluconic acid sodium salt, 75 µg of stannous chloride, and 100 µg of EDTA). Freshly eluted ^99m^Tc-pertechnetate (up to 900 MBq, 150 µL) was then added to the mixture under argon gas, and afterward, the vial was incubated at 90 °C for 60 min. The radiochemical yield was analyzed using instant thin-layer chromatography (ITLC) strips (Agilent Technologies, Santa Clara, CA, USA) eluted with PBS (Rf = 0 for the radiolabeled peptide and Rf = 1 for ^99m^Tc-pertechnetate and ^99m^Tc-gluconate) and pyridine:acetic acid:water, 5:3:1.5 (Rf = 1 for the radiolabeled peptide and Rf = 0 for reduced hydrolyzed technetium colloid). ITLC was analyzed using Cyclone Plus storage Phosphor System (PerkinElmer, Waltham, MA, USA). The reaction mixture was also analyzed on reversed-phase HPLC (Hitachi Chromaster, Luna C18 column (5 µm, 100 Å, 150 × 4.6 mm, Phenomenex, Værløse, Denmark)) with a gradient from 5 to 70% acetonitrile (0.1% *v*/*v* trifluoroacetic acid) in water over 15 min. Radio-chromatograms are shown in Supplementary files.

To evaluate the feasibility of developing a single-vial kit, the radiolabeling of maSSS-PEG_2_-RM26 was also performed using single-step labeling according to [28]. Briefly, eluate containing pertechnetate (500 MBq) was directly added to a vial containing a freeze-dried mixture of the same kit constituents and 40 µg of maSSS-PEG_2_-RM26, the vial was vortexed and afterward incubated at 90 °C for 60 min.

To test the stability, the radiolabeled peptides were incubated with 300× molar excess of cysteine or with PBS at room temperature for 1 h. The octanol-water distribution coefficient (logD) was determined as previously described [35].

### 2.2. In Vitro Assays

In Vitro Binding Specificity: To test the in vitro binding specificity of the radiolabeled peptides, PC-3 cells (5 × 10^5^ cells/well) were incubated with each radiolabeled peptide (1 nM), with or without pre-incubation with 500 nM of unlabeled peptide, at 37 °C for 1 h. After treatment with trypsin-EDTA, the detached cells were measured for their activity content.

Cellular Processing: To evaluate cellular processing of the radiolabeled peptides, PC-3 cells were incubated with the radiolabeled peptide (1 nM), and at predetermined time points, the membrane-bound and internalized fractions were collected as previously described using 4 M urea solution in 0.2 M glycine buffer (pH 2) to collect membrane-bound fraction and 1 N solution of NaOH to collect the internalized fraction [33]. Samples were measured on gamma-counter to determine activity content.

Affinity Measurements: The binding kinetics of labeled peptides was measured in real-time using LigandTracer Yellow Instruments (Ridgeview Instruments AB, Uppsala, Sweden) on PC-3 cells at room temperature, as described earlier [33]. The uptake curves were recorded at 0.5 and 2 nM of the radiolabeled peptides for 300 min. After measuring the uptake kinetics, the medium containing labeled peptide was replaced with fresh medium, and the dissociation curve was monitored over 15 h. The obtained sensorgrams were analyzed using TracerDrawer (Ridgeview Instruments AB, Uppsala, Sweden), and the dissociation constants (K_D_) were estimated.

### 2.3. In Vivo Assays

Animals: All in vivo experiments were carried out on BALB/c nu/nu or NMRI mice provided by Scanbur A/S (Sollentuna, Sweden). All animal studies were approved by the Ethics Committee for Animal Research in Uppsala (Sweden), following the national legislation on the protection of laboratory animals (project identification code of approval 5/16, 26 February 2016). Prostate cancer xenografts expressing GRPR were established in BALB/c nu/nu mice by subcutaneous injection of PC-3 cell suspension in PBS (7 × 10^6^ cells in 100 µL) four weeks before the experiment. Four to seven mice per data point were used in in vivo experiments, two mice per data point were used in metabolic analysis and SPECT/CT imaging experiments.

Biodistribution in normal mice: Biodistribution of [^99m^Tc]Tc-maSSS-PEG_2_-RM26 and [^99m^Tc]Tc-maSES-PEG_2_-RM26 and their in vivo targeting specificity to GRPR in pancreas initially was evaluated in NMRI mice 30 min post-injection (pi) of 40 pmol (30 kBq) of labelled peptide with and without co-injection of 5 nmol of unlabeled peptide. The mice were euthanized by lethal injection of ketamine/xylazine followed by exsanguination. The organs of interest were collected, weighed, and measured for their activity content on the gamma counter.

Biodistribution in tumor bearing mice: The comparison of biodistribution between the two radiolabeled peptides was evaluated in BALB/c nu/nu mice bearing PC-3 xenografts. Each mouse was injected with 40 pmol (30 kBq, mass of injected peptide was adjusted with unlabeled peptide) of either [^99m^Tc]Tc-maSSS-PEG_2_-RM26 or [^99m^Tc]Tc-maSES-PEG_2_-RM26. The mice were euthanized 3 h pi. Sample collection and activity measurements were done as described above. The biodistribution of [^99m^Tc]Tc-maSSS-PEG_2_-RM26 was further studied at 6 h pi following the same protocol. Additionally for [^99m^Tc]Tc-maSSS-PEG_2_-RM26, one group of xenograft bearing mice was injected with 40 pmol (30 kBq, mass of injected peptide was adjusted with unlabeled peptide) of labeled peptide with 5 nmol of unlabeled peptide and euthanized 3 h pi.

Metabolite Analysis: The NMRI mice were injected with 40 pmol of [^99m^Tc]Tc-maSSS-PEG_2_-RM26 (3 MBq) or [^99m^Tc]Tc-maSES-PEG_2_-RM26 (5.8 MBq). The mice were euthanized by CO_2_ inhalation 3 h pi, the content of the caecum and urine were collected and analyzed using HPLC as previously described [36]. Centrifugation was performed using Centrifuge 5430 R (Eppendorf, Hamburg, Germany).

SPECT/CT Imaging: The mice were injected with 40 pmol (3 MBq) of [^99m^Tc]Tc-maSSS-PEG_2_-RM26 alone or along with 5 nmol of maSSS-PEG_2_-RM26. The mice were imaged 3 h and 6 h pi under anesthesia; for the assessment of GRPR blocking, only imaging 3 h pi was performed.

Whole body SPECT/CT scans were performed using nanoScan SPECT/CT (Mediso Medical Imaging Systems Ltd., Budapest, Hungary). The acquisition time was 20 min. CT scans were acquired using the following parameters: X-ray energy peak of 50 keV; 670 μA; 480 projections; and 5.26 min acquisition time. SPECT raw data were reconstructed using Tera-Tomo™ 3D SPECT reconstruction technology (version 3.00.020.000; Mediso Medical Imaging Systems Ltd., Budapest, Hungary): normal dynamic range; 30 iterations; and one subset. CT data were reconstructed using Filter Back Projection in Nucline 2.03 Software (Mediso Medical Imaging Systems Ltd., Budapest, Hungary). SPECT and CT files were fused using Nucline 2.03 Software and are presented as maximum intensity projections in the RGB color scale.

Dosimetry estimation: To estimate the organs’ absorbed doses after iv injection of [^99m^Tc]Tc-maSSS-PEG_2_-RM26, NMRI mice were injected with 40 pmol (30–500 kBq) of the radiolabeled peptide and biodistribution was evaluated 0.5, 1, 3, 6, and 24 h pi. Sample collection and activity measurements were done as described above. To evaluate dosimetry in humans, uptake values in mice were upscaled using the well-established “percent kg/g method” [37] (Equation (1)):

(%IA/organ)human = [(%IA/g)animal × (kg TBweight)animal × (g organ/(kg TBweight)human](1)

where %IA-% injected activity.

Organ uptakes for human were calculated using organ weights of the reference adult male (ICRP publication 23). The data were fitted by a single exponential function, and residence time was calculated as an area under a fitted curve using Prism 8 for Windows software (GraphPad Software, San Diego, CA, USA). Absorbed doses were calculated using OLINDA/EXM 1.1 for Adult Male phantom.

### 2.4. Data Analysis

The data were analyzed using unpaired two-tailed t-tests via GraphPad Prism 8 for Windows (GraphPad Software, San Diego, CA, USA). *p*-values less than 0.05 indicated significant differences.

## 3. Results

### 3.1. Radiolabeling of Synthetic Peptides and In Vitro Characterization of [^99m^Tc]Tc-maSSS-PEG_2_-RM26 and [^99m^Tc]Tc-maSES-PEG_2_-RM26

**Figure 2:**
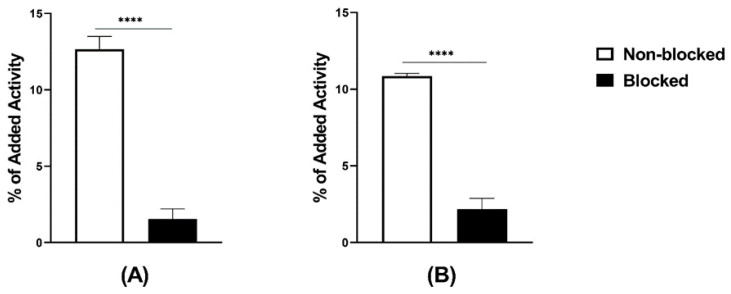
In vitro binding specificity tested on PC-3 cells, (**A**) [^99m^Tc]Tc-maSSS-PEG_2_-RM26 and (**B**) [^99m^Tc]Tc-maSES-PEG_2_-RM26 with and without prior GRPR blocking. The error bars represent the standard deviation. **** indicates a *p*-value less than 0.0001.

The radiochemical yield, determined by ITLC, was always higher than 98% for labeling following both the two-vial and the single-vial procedures. The presence of technetium colloid, determined by ITLC, was not higher than 0.2–0.3%. The radiochemical yield, determined by HPLC, was over 96% (Supplementary Figures S1A and S2A). There was no release of activity when the radiolabeled peptides were incubated with PBS, while there was a <5% release under incubation with 300× molar excess of cysteine at room temperature for 1 h. The logD values were -0.6 for [^99m^Tc]Tc-maSSS-PEG_2_-RM26 and -1.0 for [^99m^Tc]Tc-maSES-PEG_2_-RM26. Both radiolabeled peptides demonstrated specific binding to GRPR-expressing cells in vitro (Figure 2). GRPR-expressing cells that were pre-incubated with non-labeled peptides demonstrated significantly lower uptake than non-treated ones.

**Figure 3:**
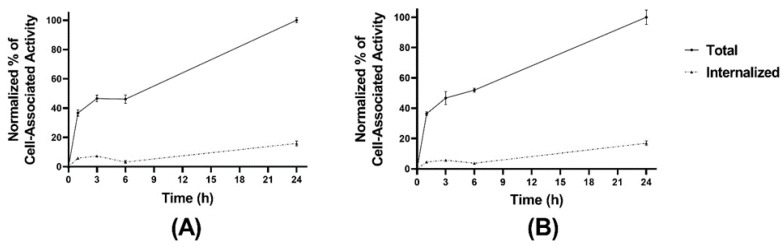
Cellular processing of [^99m^Tc]Tc-maSSS-PEG_2_-RM26 (**A**) and [^99m^Tc]Tc-maSES-PEG_2_-RM26 (**B**) at 1, 3, 6, and 24 h of incubation at 37 °C. The error bars represent the standard deviation.

**Figure 4:**
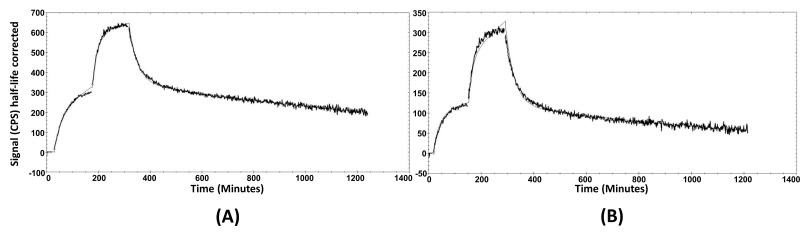
The sensorgrams obtained using LigandTracer Yellow for [^99m^Tc]Tc-maSSS-PEG_2_-RM26 (**A**) and [^99m^Tc]Tc-maSES-PEG_2_-RM26 (**B**). The concentrations during association measurements were 0.5 and 2 nM for each radiolabeled peptide.

The cellular processing of the radiolabeled peptides (Figure 3A,B) showed constantly increasing cell associated activity with slow internalization rate: 16 ± 2% of cell-associated activity for [^99m^Tc]Tc-maSSS-PEG_2_-RM26 and 17 ± 2% of [^99m^Tc]Tc-maSES-PEG_2_-RM26 after 24 h of continuous incubation were internalized. The binding kinetics of the radiolabeled peptides to GRPR were measured in real time on living cells, and the sensorgrams are shown in Figure 4A,B. The binding was fitted to a 1:2 interaction model. The dissociation constant (K_D_) was one order of magnitude higher for [^99m^Tc]Tc-maSSS-PEG_2_-RM26 than for [^99m^Tc]Tc-maSES-PEG_2_-RM26 (Table 1).

### 3.2. In Vivo Characterization

**Figure 5:**
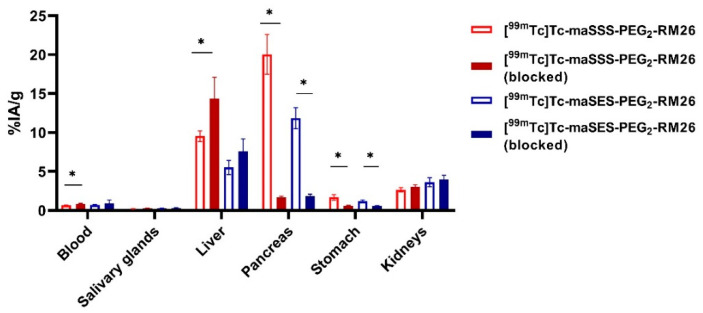
Biodistribution of activity in NMRI mice 30 min pi of either 40 pmol of [^99m^Tc]Tc-maSSS-PEG_2_-RM26 and [^99m^Tc]Tc-maSES-PEG_2_-RM26 alone or with 5 nmol of non-labeled peptide. * indicates a significant difference with a *p*-value < 0.05.

Specificity of [^99m^Tc]Tc-maSSS-PEG_2_-RM26 and [^99m^Tc]Tc-maSES-PEG_2_-RM26 to GRPR in vivo was initially demonstrated in NMRI mice by blocking receptor-mediated activity uptake in the pancreas (Figure 5). Blood clearance of both conjugates was rapid, and at 30 min pi the activity concentration in blood was below 1%IA/g (% injected activity per g of tissue). Low activity uptake in the salivary gland indicated high in vivo stability of the Tc-99m label for both peptides. Groups co-injected with a high dose of non-labeled peptide demonstrated significantly lower uptake in pancreas and stomach (*p* < 0.001), organs with endogenous GRPR expression. It should be mentioned that activity uptake in the liver was higher in the blocked group (significantly higher for [^99m^Tc]Tc-maSSS-PEG_2_-RM26).

**Figure 6:**
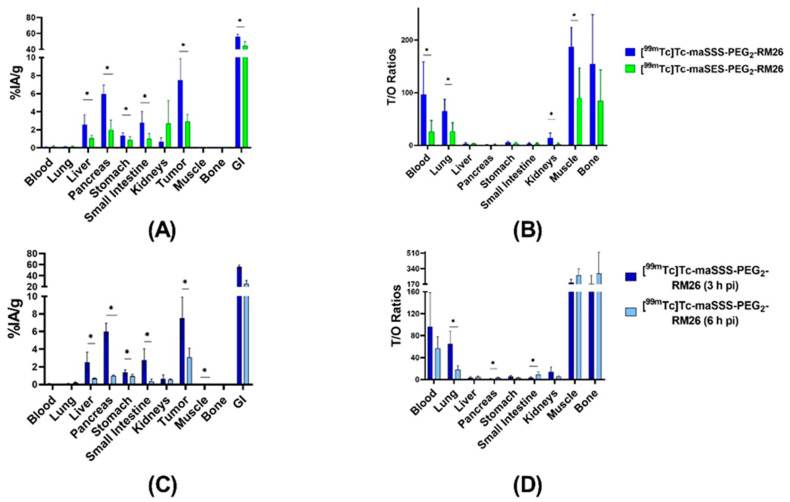
Biodistribution of [^99m^Tc]Tc-maSSS-PEG_2_-RM26 and [^99m^Tc]Tc-maSES-PEG_2_-RM26 in BALB/C nu/nu mice bearing PC-3 xenografts: The biodistribution (**A**) and the tumor-to-organ ratios (**B**) for [^99m^Tc]Tc-maSSS-PEG_2_-RM26 (blue) and [^99m^Tc]Tc-maSES-PEG_2_-RM26 (green) at 3 h pi. The biodistribution (**C**) and the tumor-to-organ ratios (**D**) for [^99m^Tc]Tc-maSSS-PEG_2_-RM26 at 3 h pi (dark blue) and 6 h pi (light blue). The uptake in the gastrointestinal tract with content (GI) is presented as %IA. The error bars represent the standard deviation. * indicates a significant difference with a *p*-value < 0.05.

The biodistribution of [^99m^Tc]Tc-maSSS-PEG_2_-RM26 and [^99m^Tc]Tc-maSES-PEG_2_-RM26 was compared in xenografted mice 3 h pi (Figure 6A and Supplementary Table S1). The biodistribution of [^99m^Tc]Tc-maSES-PEG_2_-RM26 demonstrated remarkably lower activity uptake than [^99m^Tc]Tc-maSSS-PEG_2_-RM26 in most normal organs, except kidneys. However, activity uptake was more than 2-fold higher in tumors for [^99m^Tc]Tc-maSSS-PEG_2_-RM26 with 7 ± 2%IA/g vs 2.9 ± 0.7%IA/g. The activity uptake in the gastrointestinal tract (with content) was high for both conjugates, over 50%IA, despite moderate uptake in livers 3 h pi. The tumor-to-non-tumor ratios for [^99m^Tc]Tc-maSSS-PEG_2_-RM26 were higher than for [^99m^Tc]Tc-maSES-PEG_2_-RM26 (Figure 6B and Supplementary Table S1), with tumor-to-blood ratios of 97 ± 56 for [^99m^Tc]Tc-maSSS-PEG_2_-RM26 and 26 ± 19 for [^99m^Tc]Tc-maSES-PEG_2_-RM26, tumor-to-muscle ratios of 188 ± 32 and 90 ± 52, and tumor-to-bone ratios of 177 ± 79 and 84 ± 54, respectively.

The biodistribution of [^99m^Tc]Tc-maSSS-PEG_2_-RM26 6 h pi (Figure 6C and Supplementary Table S1) showed decreased uptake in all organs compared with 3 h. The uptake in tumor decreased 2-fold and was 3 ± 1%IA/g. The activity uptake in the gastrointestinal tract also decreased 2-fold. This resulted in almost equal tumor-to-organ ratios for 3 h and 6 h pi (Figure 6D and Supplementary Table S1).

**Figure 7:**
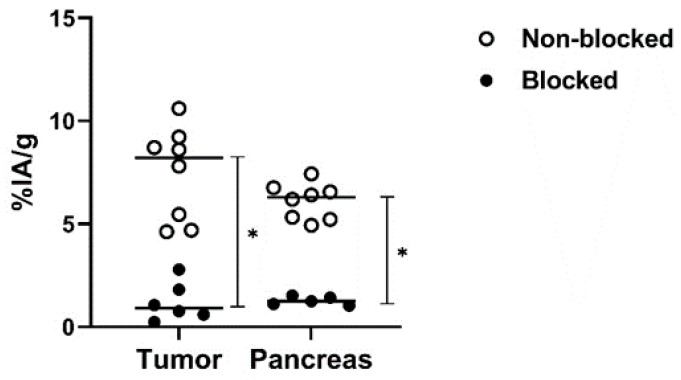
The dot plot representing the in vivo GRPR targeting specificity for [^99m^Tc]Tc-maSSS-PEG_2_-RM26 on BALB/C nu/nu mice bearing PC-3 xenografts at 30 min pi. Each white dot denotes the %IA/g of tissue uptake in a mouse injected with [^99m^Tc]Tc-maSSS-PEG_2_-RM26 alone. Each black dot denotes the %IA/g of tissue uptake in a mouse injected with [^99m^Tc]Tc-maSSS-PEG_2_-RM26 along with 5 nmol of maSSS-PEG_2_-RM26. * indicates a significant difference with a *p*-value < 0.05.

The in vivo specificity experiment (Figure 7) demonstrated significantly lower activity uptake of [^99m^Tc]Tc-maSSS-PEG_2_-RM26 in PC-3 tumors and pancreas when 5 nmol of maSSS-PEG_2_-RM26 were co-injected compared with injection of [^99m^Tc]Tc-maSSS-PEG_2_-RM26 at low peptide dose, reflecting specific binding of [^99m^Tc]Tc-maSSS-PEG_2_-RM26 to GRPR in vivo.

### 3.3. Metabolite Analysis

Analysis of the urine and extract of the gastrointestinal (GI) tract content on radio HPLC revealed no peaks corresponding to the intact peptide. Instead, hydrophilic metabolites were observed at earlier retention times; the chromatograms are shown in the Supplementary file (Figures S1B,C and S2B,C).

### 3.4. SPECT/CT Imaging

**Figure 8:**
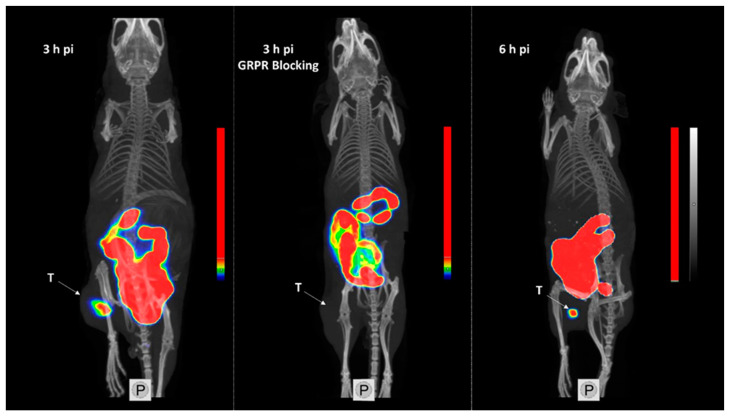
Single-photon emission computed tomography/computed tomography (SPECT/CT) images of BALB/C nu/nu mice bearing PC-3 xenografts (denoted by a letter T) injected with [^99m^Tc]Tc-maSSS-PEG_2_-RM26 at 3 and 6 h pi, or with [^99m^Tc]Tc-maSSS-PEG_2_-RM26 in addition to 5 nmol of maSSS-PEG_2_-RM26 to block GRPR.

The obtained SPECT/CT images correlated to the biodistribution profile for [^99m^Tc]Tc-maSSS-PEG_2_-RM26 3 and 6 h pi (Figure 8). Tumors were clearly visualized. An elevated uptake in the GI was also clearly visible. No activity uptake was detected in the liver. When GRPR in xenografts was blocked by co-injection of non-labeled maSSS-PEG_2_-RM26, no uptake of [^99m^Tc]Tc-maSSS-PEG_2_-RM26 in PC-3 tumor was observed. 

### 3.5. Dosimetry Estimations

Absorbed doses for men estimated from ex vivo data for mice (Supplementary Table S2) are presented in Table 2. The highest absorbed organ dose was in the small intestines (1.65 × 10^−2^ mGy/MBq) followed by the upper large intestine wall (ULI wall, 1.06 × 10^−2^ mGy/MBq). Doses to other normal organs and tissues were below 10^−2^ mGy/MBq. The total effective dose was 0.0035 mSv/MBq.

## 4. Discussion

The identification of GRPR as an important target for imaging and treatment of prostate cancers has led to the development of numerous GRPR-targeting radiotracers with the aim of improving the sensitivity for detecting prostate cancer and its distant metastases. In this study, we designed and preclinically evaluated two new GRPR antagonists for SPECT imaging using the widely available gamma-emitter technetium-99m. The peptides were designed to have amino acid containing N_3_S chelators to provide a convenient labeling method with ^99m^Tc. 

The labeling of both peptides with ^99m^Tc was successful with high radiochemical yields. Moreover, the one-pot single-step labeling of maSSS-PEG_2_-RM26 using the kit approach [28] was performed with high radiochemical yields. This facilitates its utility in clinics by minimizing handling of activity and exclusion further purification. Targeting properties of labeled peptides were further investigated in vitro an in vivo using standard methodology [25,38]. Both radiolabeled peptides, [^99m^Tc]Tc-maSSS-PEG_2_-RM26 and [^99m^Tc]Tc-maSES-PEG_2_-RM26, despite the addition of four amino acids to the parental nona-peptide, bound specifically to GRPR in PC-3 cells with subnanomolar affinity and retained their GRPR antagonistic property, recognizable by the slow internalization in PC-3 cells (see Figure 3 and Figure 4 and Table 1). The binding specificity of tested peptides was demonstrated both in vitro (Figure 2) and in vivo (Figure 5, Figure 6, Figure 7 and Figure 8) by blocking receptors with an excess of non-labeled peptides. This corroborates published data that the binding affinity of bombesin analogs is less sensitive to modifications incorporated at N-termini [39,40]. 

As hypothesized, the incorporation of glutamic acid in maSES-PEG_2_-RM26 resulted in increased hydrophilicity; this was well-reflected in the octanol-water distribution coefficient values. The increased hydrophilicity was also evident in the biodistribution profiles of peptides, shown in Figure 6A, with a significantly lower hepatic uptake of [^99m^Tc]Tc-maSES-PEG_2_-RM26 than [^99m^Tc]Tc-maSSS-PEG_2_-RM26. Additionally, there was a tendency, although not statistically significant, for higher kidney uptake of [^99m^Tc]Tc-maSES-PEG_2_-RM26 compared with [^99m^Tc]Tc-maSSS-PEG_2_-RM26. However, the incorporation of glutamic acid negatively influenced the GRPR targeting for [^99m^Tc]Tc-maSES-PEG_2_-RM26. This was observed in the affinity measurements of these peptides, with the dissociation constant for [^99m^Tc]Tc-maSES-PEG_2_-RM26 being at least one order of magnitude lower than for [^99m^Tc]Tc-maSSS-PEG_2_-RM26 (849 pM vs 61 pM). This moderate drop in affinity was expected because it is known that bombesin analogs with positive charges at their N-termini exhibit better affinities for GRPR than those containing negative charges [39]. However, it should be noticed that [^99m^Tc]Tc-maSES-PEG_2_-RM26 still has affinity to GRPR in the subnanomolar range (Table 1). Therefore, both variants were further evaluated in vivo. It is interesting to note that binding curves (Figure 4A,B) for RM26 labeled with technetium-99m via amino acids had the best fitting to 1:2 interaction model, but not to 1:1 model that could be expected for such a short peptide. This could reflect two different conformations of labeled peptide. 

The compromised affinity towards GRPR resulted in significantly lower [^99m^Tc]Tc-maSES-PEG_2_-RM26 uptake in the pancreas, which has endogenous expression of GRPR [1,41], and the GRPR-expressing PC-3 xenografts than [^99m^Tc]Tc-maSSS-PEG_2_-RM26. The higher tumor uptake of [^99m^Tc]Tc-maSSS-PEG_2_-RM26 resulted in overall higher tumor-to-organ ratios for [^99m^Tc]Tc-maSSS-PEG_2_-RM26 than for [^99m^Tc]Tc-maSES-PEG_2_-RM26, which should translate into images with higher contrast. The comparisons between [^99m^Tc]Tc-maSSS-PEG_2_-RM26 and [^99m^Tc]Tc-maSES-PEG_2_-RM26 led to the selection of [^99m^Tc]Tc-maSSS-PEG_2_-RM26 for further evaluation in vivo. The biodistribution of [^99m^Tc]Tc-maSSS-PEG_2_-RM26 at a later time point (6 h pi) demonstrated an appreciable decrease in activity uptake in all studied tissues. However, the tumor-to-organ ratios were higher at 3 h pi because of the 2-fold decrease in tumor activity uptake between 3 h and 6 h pi. Therefore, we conclude that [^99m^Tc]Tc-maSSS-PEG_2_-RM26 can be used for imaging of GRPR-overexpression in prostate cancer in clinics a few hours after injection. This also would provide shorter waiting times for patients, and less injected activity would be needed to acquire the images. Determination of optimal time for imaging could be done when human data will be available.

A comparison in terms of ex vivo tumor-to-organ ratios between [^99m^Tc]Tc-maSSS-PEG_2_-RM26 performance 3 h pi and other reported ^99m^Tc-labelled GRPR antagonists at this time point showed comparable tumor-to-blood, tumor-to-liver, tumor-to-kidney, tumor-to-intestines and tumor-to-muscle ratios for [^99m^Tc]Tc-maSSS-PEG_2_-RM26 and [^99m^Tc]Tc-Demobesin 1, [^99m^Tc]Tc-4 [36], [^99m^Tc]Tc-DB7, [^99m^Tc]Tc-DB13 and [^99m^Tc]Tc-DB14 [42], with the exception of a two-fold higher tumor-to-muscle ratio for [^99m^Tc]Tc-Demobesin 1. [^99m^Tc]Tc-maSSS-PEG_2_-RM26 also had tumor-to-blood and tumor-to-kidneys ratios close to [^99m^Tc]-N4-AR and [^99m^Tc]-N4-BB-ANT [25,43]. However, both [^99m^Tc]Tc-N4-AR and [^99m^Tc]Tc-N4-BB-ANT had higher tumor-to-liver, tumor-to-intestines and tumor-to-muscle ratios. Planned clinical study would provide data for further comparison of the developed GRPR-targeting agent with other GRPR-targeting agents labeled with technetium-99m.

High hepatobiliary uptake activity of [^99m^Tc]Tc-maSSS-PEG_2_-RM26 led to high activity excretion into the gastrointestinal content. The metabolite analysis of samples obtained 3 h pi from the gastrointestinal tract and urine showed no intact radiolabeled peptide, which indicates metabolic degradation of imaging probe in liver and kidneys that accompanied the excretion. The acquired SPECT/CT images indicated clear detection of the implanted GRPR-expressing tumors despite the high uptake in the intestines content, a pattern also seen to different extents for the other ^99m^Tc-labeled GRPR antagonists [36,42]. The elevated hepatobiliary uptake may not be of great concern when imaging prostate cancer in clinics, mainly because the tissues of interest or anatomical context are muscle and low large intestinal walls, and to a lesser extent, bones. Moreover, it might be even favorable, as it decreases activity in the bladder, which interferes with imaging of oligometastatic disease. It has to be taken into account that the process of hepatobiliary excretion in humans is much slower than in rodents, and therefore, the observed hepatobiliary excretion would not be critical for imaging of local lymph node metastases.

The dosimetry estimations for [^99m^Tc]Tc-maSSS-PEG_2_-RM26 were in low values of mGy/MBq, predicting low absorbed doses to organs and an effective dose of 3.49 × 10^−3^ mSv/MBq, as shown in Table 1. The dosimetry calculations are in the same range with dosimetry based on clinical data for other ^99m^Tc-labeled GRPR targeting agents such as [^99m^Tc]Tc-RP527 and [^99m^Tc]Tc-Demobesin 4 [44,45] with higher doses of activity to intestines but a lower effective dose than either mentioned radiolabeled GRPR agonist.

## 5. Conclusions

This study presents the development and pre-clinical characterization of a new promising GRPR antagonist that can be easily radiolabeled with technetium-99m through single-step labeling. The radiotracer can be used to image GRPR-overexpressing malignancies, with prostate cancer being the focus, utilizing the wide availability of ^99m^Tc and SPECT worldwide. Clinical translation of the findings herein will be sought and evaluated.

## Figures and Tables

**Table 1:** Affinity measurements of [^99m^Tc]Tc-maSSS-PEG_2_-RM26 and [^99m^Tc]Tc-maSES-PEG_2_-RM26.

Interaction Constants	[^99m^Tc]Tc-maSSS-PEG_2_-RM26	[^99m^Tc]Tc-maSES-PEG_2_-RM26
k_a_1 (M^−1^sec^−1^)	1.67 × 10^5^	1.63 × 10^4^
k_d_1 (sec^−1^)	1.02 × 10^−5^	1.38 × 10^−5^
K_D_1 (pM)	61	849
k_a_2 (M^−1^sec^−1^)	4.22 × 10^5^	3.72 × 10^5^
k_d_2 (sec^−1^)	5.04 × 10^−4^	5.09 × 10^−4^
K_D_2 (nM)	1.2	1.4

k_a_-asociation constant, k_d_-dissociation constant, K_D_-equilibrium dissociation constant.

**Table 2:** Calculated absorbed dose (mGy/MBq) for [^99m^Tc]Tc-maSSS-PEG_2_-RM26 in humans using OLINDA/EXM 1.1.

Target Organ	Total, mGy/MBq
Adrenals	1.12 × 10^−3^
Brain	7.43 × 10^−5^
Breasts	3.01 × 10^−4^
Gallbladder wall	3.02 × 10^−3^
LLI * wall	7.31 × 10^−3^
Small intestine	1.65 × 10^−2^
Stomach wall	7.69 × 10^−3^
ULI * wall	1.06 × 10^−2^
Heart wall	2.37 × 10^−3^
Kidneys	2.09 × 10^−3^
Liver	2.61 × 10^−3^
Lungs	6.15 × 10^−4^
Muscle	7.97 × 10^−4^
Pancreas	3.59 × 10^−3^
Red marrow	1.12 × 10^−3^
Osteogenic cells	1.60 × 10^−3^
Skin	2.97 × 10^−4^
Spleen	1.74 × 10^−3^
Testes	3.10 × 10^−4^
Thymus	4.66 × 10^−4^
Thyroid	1.89 × 10^−4^
Urinary bladder wall	1.26 × 10^−3^
Total body	1.11 × 10^−3^
Effective dose equivalent (mSv/MBq)	4.19 × 10^−3^
Effective dose (mSv/MBq)	3.49 × 10^−3^

* ULI—upper large intestine, LLI—lower large intestine.

## Data Availability

Data is contained within the article or supplementary material.

## Supplementary Materials

The following are available online at https://www.mdpi.com/1999-4923/13/2/182/s1, Figure S1: (A) Radio-chromatogram of [^99m^Tc]Tc-maSSS-PEG_2_-RM26 before injection in NMRI mice. (B) Radio-chromatogram of the collected urine samples from two mice 3 h after injecting [^99m^Tc]Tc-maSSS-PEG_2_-RM26. (C) Radio-chromatogram of the collected caecum content from two mice 3 h after injecting [^99m^Tc]Tc-maSSS-PEG_2_-RM26; Figure S2: (A) Radio-chromatogram of [^99m^Tc]Tc-maSES-PEG_2_-RM26 before injection in NMRI mice. (B) Radio-chromatogram of the collected urine samples from two mice 3 h after injecting [^99m^Tc]Tc-maSES-PEG_2_-RM26. (C) Radio-chromatogram of the collected caecum content from two mice 3 h after injecting [^99m^Tc]Tc-maSES-PEG_2_-RM26; Table S1: The biodistribution data of [^99m^Tc]Tc-maSES-PEG_2_-RM26 and [^99m^Tc]Tc-maSSS-PEG_2_-RM26: Table S2: The biodistribution data for [^99m^Tc]Tc-maSSS-PEG_2_-RM26 in NMRI mice.
