# Supplementary material for: Preclinical Evaluation of 99mTc-Labeled GRPR Antagonists maSSS/SES-PEG2-RM26 for Imaging of Prostate Cancer
Source: Pharmaceutics. 2021 Jan 30;13(2):182. doi: 10.3390/pharmaceutics13020182 (PMC7912279; doi:10.3390/pharmaceutics13020182)
Supplement: Supplementary file 1 [file pharmaceutics-13-00182-s001.pdf]

pharmaceutics

MDPI

Supplementary Materials: Preclinical Evaluation of ^{99m}Tc-Labeled GRPR Antagonists maSSS/SES-PEG2-RM26 for Imaging of Prostate Cancer

Ayman Abouzayed, Sara S. Rinne, Hamideh Sabahnoo, Jens Sörensen, Vladimir Chernov, Vladimir Tolmachev and Anna Orlova

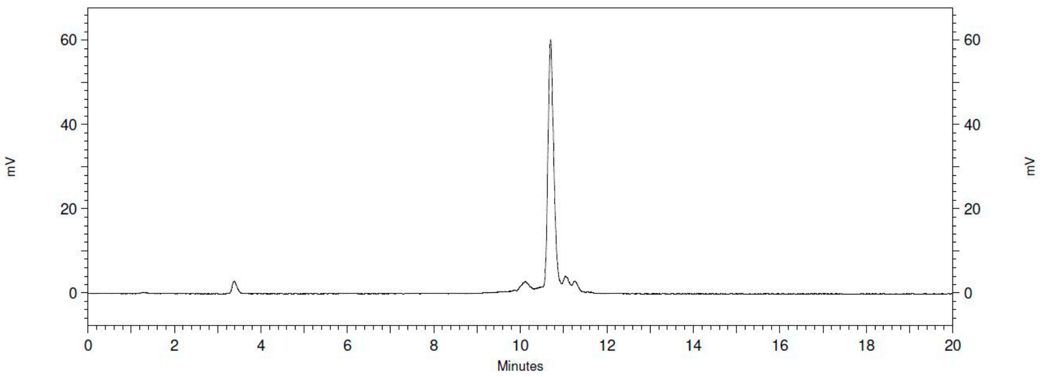

(**A**)

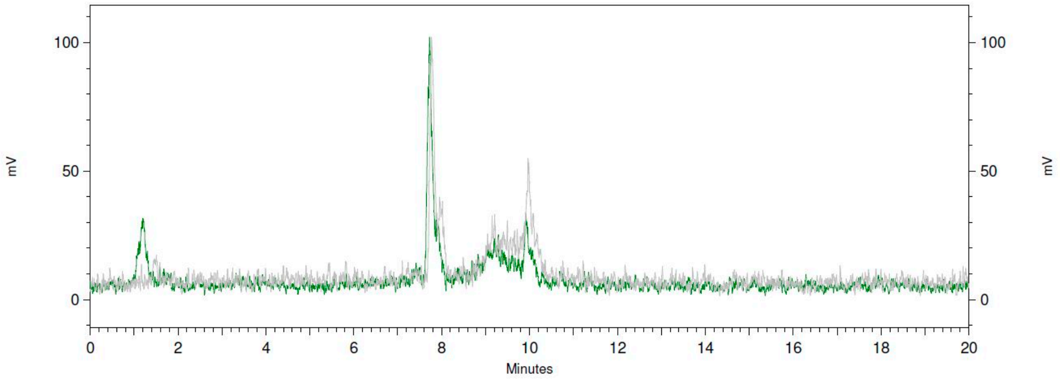

(**B**)

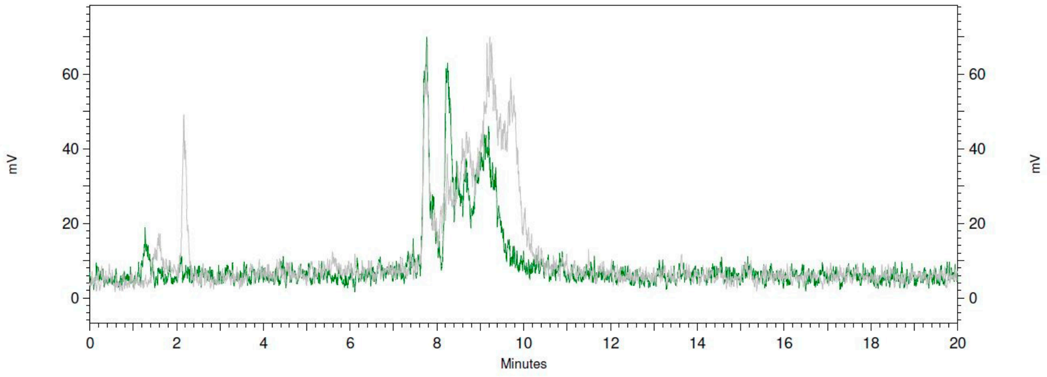

(**C**)

Figure S1. (**A**) Radio-chromatogram of [^{99m}Tc]Tc-maSSS-PEG$_2$-RM26 before injection in NMRI mice. (**B**) Radio-chromatogram of the collected urine samples from two mice 3 h after injecting [^{99m}Tc]Tc-maSSS-PEG$_2$-RM26. (**C**) Radio-chromatogram of the collected caecum content from two mice 3 h after injecting [^{99m}Tc]Tc-maSSS-PEG$_2$-RM26. The x-axes show the retention time in minutes and the y-axes show the signal.

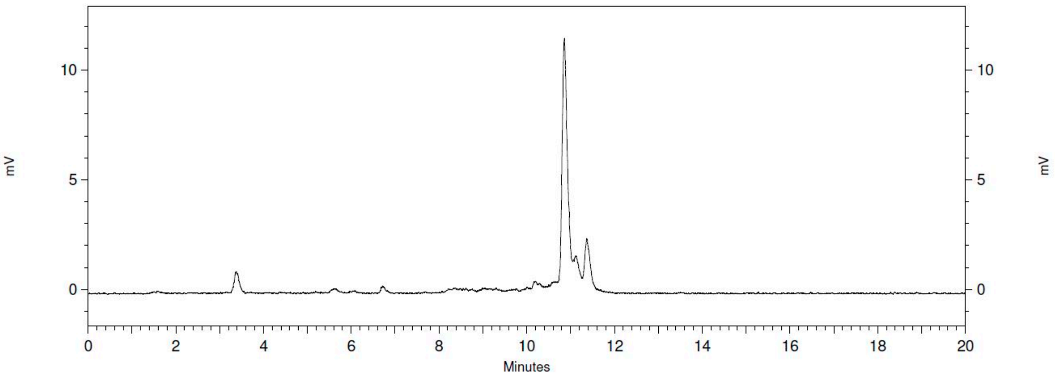

(**A**)

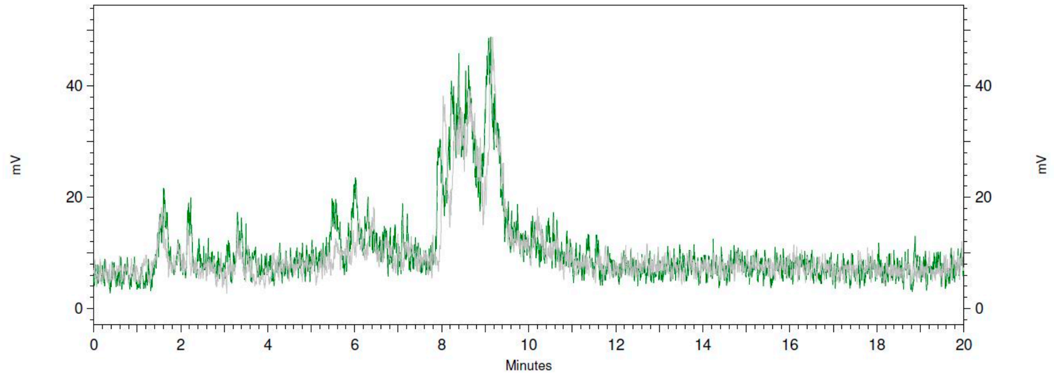

(**B**)

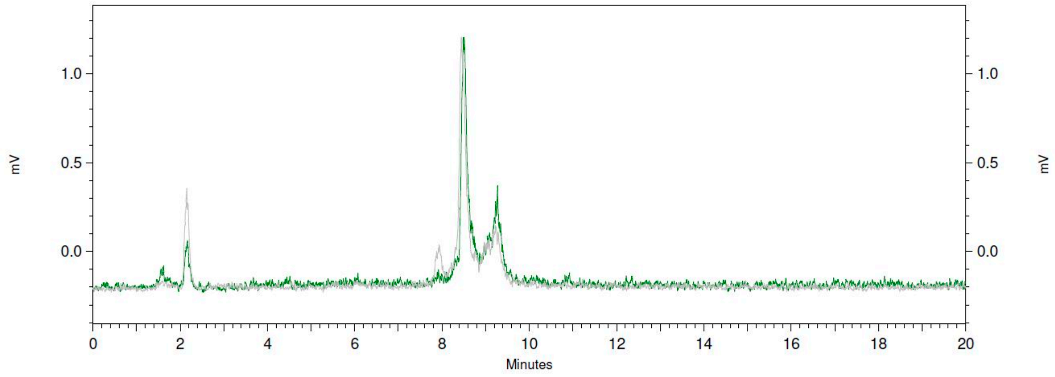

(**C**)

Figure S2. (**A**) Radio-chromatogram of [^{99m}Tc]Tc-maSES-PEG$_2$-RM26 before injection in NMRI mice. (**B**) Radio-chromatogram of the collected urine samples from two mice 3 h after injecting [^{99m}Tc]Tc-maSES-PEG$_2$-RM26.(**C**) Radio-chromatogram of the collected caecum content from two mice 3 h after injecting [^{99m}Tc]Tc-maSES-PEG$_2$-RM26. The x-axes show the retention time in minutes and the y-axes show the signal.

Table S1. The biodistribution data for [^{99m}Tc]Tc-maSES-RM26 and [^{99m}Tc]Tc-maSSS-RM26 in Balb/c nu/nu mice bearing PC-3 xenografts.

**Conjugate**	**[^{99m}Tc]Tc-maSES-RM26**	**[^{99m}Tc]Tc-maSSS-RM26**	
**Elapsed time**	**3 h**	**3 h**	**6 h**
**Organs**	**% IA/g**		
Blood	0.15 ± 0.07	0.09 ± 0.04	0.056 ± 0.009
Lung	0.14 ± 0.07	0.12 ± 0.03	0.20 ± 0.10
Liver	1.1 ± 0.3 *	3 ± 1	0.68 ± 0.08 †
Spleen	0.11 ± 0.02	0.12 ± 0.04	0.06 ± 0.01 †
Pancreas	2 ± 1 *	6.1 ± 0.8	0.98 ± 0.08 †
Stomach	0.9 ± 0.4 *	1.4 ± 0.3	0.9 ± 0.2 †
Intestine	1.1 ± 0.6 *	3 ± 1	0.4 ± 0.2 †
Kidneys	3 ± 2	0.7 ± 0.4	0.59 ± 0.06
Tumor	2.9 ± 0.7 *	7 ± 2	3 ± 1 †
Muscle	0.04 ± 0.03	0.04 ± 0.02	0.012 ± 0.004 †
Bone	0.04 ± 0.02	0.04 ± 0.02	0.02 ± 0.02
GI	46 ± 4 *‡	56 ± 3 ‡	26 ± 6 †‡
	**Tumor-to-non-tumor ratio**		
Blood	26 ± 19 *	97 ± 56	57 ± 22
Lung	26 ± 16 *	65 ± 20	18 ± 8 †
Liver	3.0 ± 0.8	3 ± 2	5 ± 2
Spleen	24 ± 10 *	62 ± 14	50 ± 16
Pancreas	1.5 ± 0.8	1.2 ± 0.4	3 ± 1 †
Stomach	4 ± 2	5 ± 2	3 ± 1
Intestine	3 ± 2	3 ± 1	9 ± 4 †
Kidneys	2 ± 2 *	14 ± 9	5 ± 2
Muscle	90 ± 52 *	188 ± 32	270 ± 67
Bone	84 ± 54	177 ± 79	287 ± 232

*Significant difference between the values for [^{99m}Tc]Tc-maSES-RM26 and for [^{99m}Tc]Tc-maSSS-RM26 at 3 h pi. †Significant difference between the values for [^{99m}Tc]Tc-maSSS-RM26 at 6 h pi and at 3 h pi. ‡The value calculated as the % IA.

Table S2. The biodistribution data for [^{99m}Tc]Tc-maSSS-RM26 in NMRI mice.

**Elapsed time**	**0.5 h**	**1 h**	**3 h**	**6 h**	**24 h**
**Organs**			**% IA/g**		
Blood	0.7 ± 0.1	0.8 ± 0.1	0.38 ± 0.06	0.30 ± 0.07	0.030 ± 0.006
Heart	0.27 ± 0.10	0.23 ± 0.03	0.12 ± 0.01	0.17 ± 0.08	0.021 ± 0.003
Lung	0.7 ± 0.3	0.9 ± 0.4	0.44 ± 0.07	0.3 ± 0.1	0.09 ± 0.04
Liver	4 ± 1	6 ± 1	2.5 ± 0.4	1.1 ± 0.3	0.39 ± 0.05
Spleen	1.1 ± 0.4	2.4 ± 1.6	2 ± 1	0.6 ± 0.5	0.37 ± 0.09
Pancreas	7 ± 2	13 ± 6	3.2 ± 0.7	0.8 ± 0.2	0.10 ± 0.02
Kidneys	2.5 ± 0.6	4 ± 1	1.5 ± 0.3	1.3 ± 0.3	0.46 ± 0.08
Muscle	0.13 ± 0.03	0.12 ± 0,02	0.061 ± 0.006	0.05 ± 0.03	0.006 ± 0.003
Bone	0.26 ± 0.09	0.2 ± 0.1	0.27 ± 0.04	0.18 ± 0.03	0.04 ± 0.02
Brain	0.031 ± 0.008	0.04 ± 0.02	0.03 ± 0.01	0.03 ± 0.01	0.006 ± 0.004
			%IA/sample		
Stomach	2.2 ± 0.2	6 ± 2	5 ± 1	3.2 ±0.4	0.2 ± 0.1
Small intestine	10 ± 2	44 ± 10	9 ± 3	2.5 ±0.8	0.10 ± 0.05
Upper large intestine	0.4 ± 0.3	0.8 ± 0.5	5.2 ± 5.1	14 ± 1	0.05 ± 0.01
Large intestine	0. 7 ± 0.8	0.6 ± 0.1	3.3 ± 3.5	30 ± 13	0.05 ± 0.03
Caecum	0.34 ± 0.09	0.9 ± 0.2	16 ± 4	18 ± 3	0.3 ± 0.2
Body	4.5 ± 0.9	6 ± 1	4 ± 2	2.8 ± 0.2	0.27 ± 0.05
